# Under five and infant mortality in Chile (1990-2016): Trends, disparities, and causes of death

**DOI:** 10.1371/journal.pone.0239974

**Published:** 2020-09-30

**Authors:** Ximena Aguilera, Iris Delgado, Gloria Icaza, Mauricio Apablaza, Loreto Villanueva, Carla Castillo-Laborde

**Affiliations:** 1 Centro de Epidemiología y Políticas de Salud (CEPS), Facultad de Medicina, Universidad del Desarrollo, Santiago, Chile; 2 Programa de Investigación Asociativa de Cáncer Gástrico, Instituto de Matemática y Física, Universidad de Talca, Talca, Chile; 3 Facultad de Gobierno, Universidad del Desarrollo, Santiago, Chile; 4 Oxford Poverty and Human Development Innitiative (OPHI), University of Oxford, Oxford, United Kingdom; 5 Departamento de Promoción de la Salud de la Mujer y el Recién Nacido, Universidad de Chile, Santiago, Chile; Leibniz Institute for Prevention Research and Epidemiology BIPS, GERMANY

## Abstract

**Background:**

Child health has been a health policy priority for more than a century in Chile. Since 2000, new health and intersectoral interventions have been implemented. However, no recent analyses have explored child mortality and equity in Chile, an indispensable input to guide policies towards the achievement of the Sustainable Development Goals, specially, in the context of a deeply unequal country such as many other Latin American countries. Thus, the objectives of this study are to analyze the variations in the risk and the causes of death among Chilean children aged <5 years, to identify the determinants, and to measure inequality of infant mortality from 1990 to 2016.

**Materials and methods:**

An observational study was conducted to analyze the Chilean children's mortality from 1990 to 2016 using under five deaths and live births data from the Vital Statistics System. To describe the variation in the risk of death, a time series analysis was performed for each of the under five mortality rate components. A comparative cause of death analysis was developed for Neonatal and 1–59 months’ age groups. The determinants of infant mortality were studied with a descriptive analysis of yearly rates according to mother’s and child factors and bivariate logistic regression models at the individual level. Finally, simple and complex measures of inequality at individual level were estimated considering three-year periods.

**Results:**

Regarding under 5 mortality: (i) Child survival has improved substantially in the last three decades, with a rapid decline in under five mortality rate between 1990 and 2001, followed by a slower reduction; (ii) early neonatal mortality has become the main component of the under five mortality rate (50.6%); (iii) congenital abnormalities have positioned as the leading cause of death; (iv) an important increase in live births below 1,000 grs. Regarding infant mortality: (i) birth weight and gestational age are the two most relevant risk factors in the neonatal period, while social variables are more significant for post-neonatal mortality and, (ii) the inequality according to mother’s education has shown a steady decline, with persistent inequalities in post-neonatal period.

**Conclusions:**

The Chilean experience illustrates child health achievements and challenges in a country that transitioned from middle-to high-income in recent decades. Although inequity is one of the main challenges for the country, the health sector by granting universal access was able to reduce disparities. However, closing the gap in post-neonatal mortality is still challenging. To overcome stagnation in neonatal mortality, new and specific strategies must address current priorities, emphasizing the access of vulnerable groups.

## Introduction

Economic development, together with expanded access to health care, has brought about a significant reduction in childhood mortality in recent decades worldwide [1]. However, inequality stigmas persist; child mortality in low-income families almost duplicates the risk of childhood death among the wealthiest, and children born to mothers with no education have significantly lower survival rates than those born to mothers with secondary or higher education [2]. Under the slogan "leaving no one behind”, the 2030 Agenda of the United Nations has emphasized the importance of equity in achieving Sustainable Development Goals (SDGs). The goal for child health by 2030 (SDG 3.2) is to reduce preventable neonatal and under five children´s deaths to rates below 12 and 25 deaths per 1,000 births, respectively. Promoting intersectoral action on social determinants and improving access to health care are the main strategies to reach these goals [3, 4].

Achieving both goals, reducing child deaths, and tackling inequalities is a challenging target for all Latin American countries. In Chile, child health has been a priority and a stated objective for health policy for more than a century; and, significant improvements have been achieved, especially in the second half of the last century [5–9].

Although there has been a sustained reduction of the IMR globally in the last decade [1, 2], in Chile, we observed a relative stagnation in spite of governmental strategies to expand access to health care and intersectoral actions to address the social determinants of child health [8–15]. Jointly with Mexico and Turkey, Chile is one of the few high-income countries with an IMR over five deaths per 1,000 live births [16]. The associated factors of this IMR inertia and their effects in inequality are not fully understood. Additionally, no recent analyses of the inequalities of child mortality or their evolution are available. Such analyses are an indispensable guide for developing policies to achieve the SDGs in child health without leaving anyone behind. This allow us to identify current health priorities regarding the new vulnerable groups and emerging causes of child death—including crucial social determinants—to design targeted public policies in a highly unequal context.

Using a unique dataset of complete and reliable administrative records from the Chilean National Vital Statistics System, our aim is to elucidate the factors affecting under five and infant mortality trends in Chile. The objectives of this paper are to analyze the variations in the risk of death in children aged <5 years and to describe changes in the patterns of the cause of death between 1990 and 2016. Additionally, the paper identifies determinants of infant mortality and explores its inequality evolution during this period using simple and complex indicators.

## Materials and methods

An observational study was conducted to analyze Chilean children's mortality from 1990 to 2016 in a comprehensive manner to address the objective and its various perspectives, as stated previously. The study includes analyses of trends, causes of death, mortality determinants and, socioeconomic inequality.

### Data and sources

The under five deaths and live births data (1990–2016) are found in administrative records from Chile´s Vital Statistics Systems. These records are considered to be in the best performance category globally with a broad coverage of recording close to 100% [17]. Records of live births and deaths include demographic (sex, age) and geographic (region and municipality of residence) information. Data for live births and deaths of infants less than one-year-old include a set of additional sociodemographic variables with parent´s information (parent´s age, marital status, schooling, occupational activity), as well as characteristics of the birth, such as weight, length, and gestational age. The father´s information was missing for more than 30% of the cases and was therefore excluded from the analysis.

### Definition of variables

**Fig 1:**
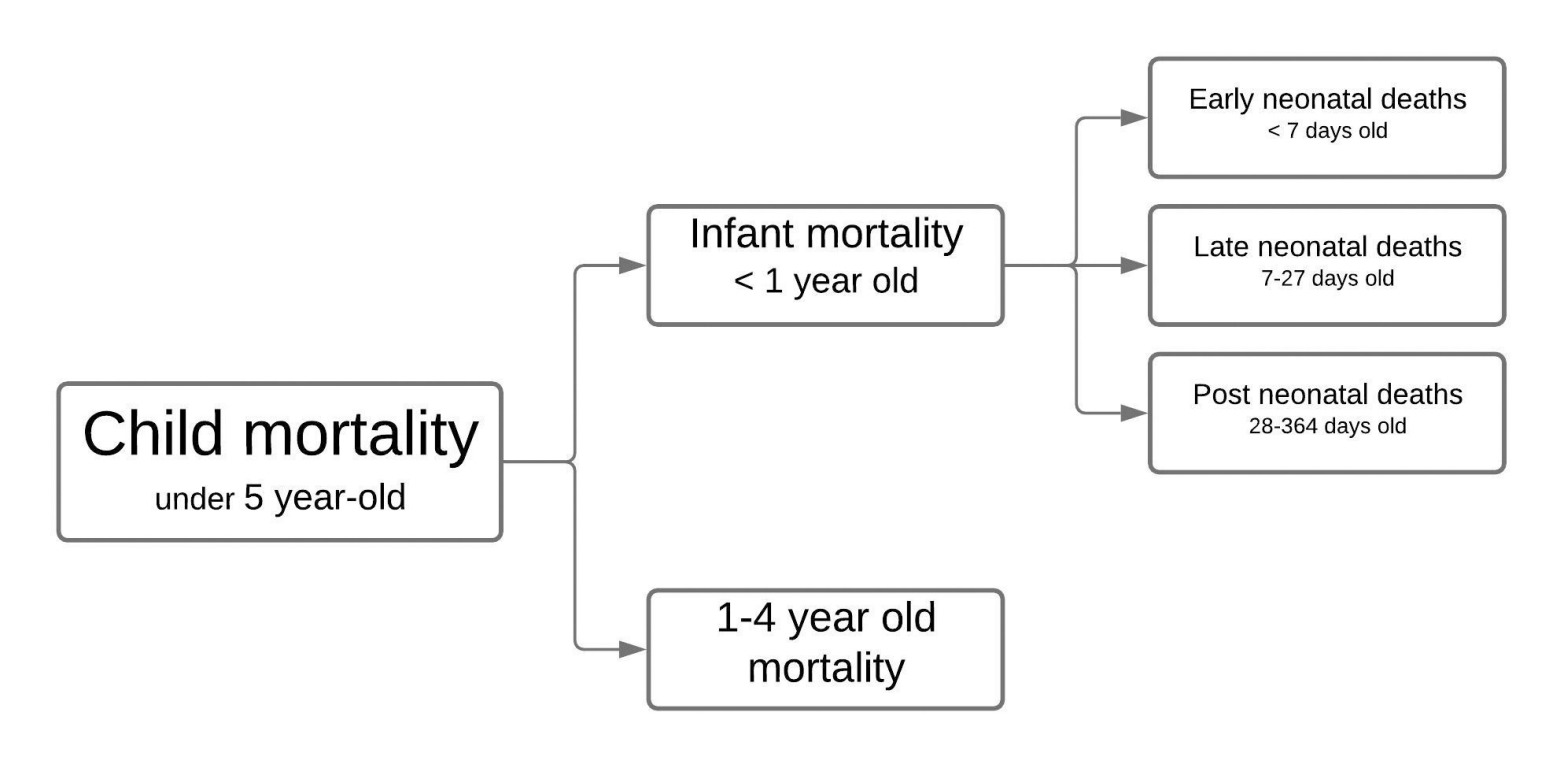
Child mortality components.

The dependent variable, child mortality, was broken down into specific mortality rates: under five; infant (< 1 year); early and late neonatal (<7 days and 7–27 days, respectively); post-neonatal (28–364 days); and 1- to 4-year-old children (Fig 1).

Independent variables include maternal and child factors and they were categorized as follows: mother´s age (<20; 20 to 39; +40 years); marital status (single; married); occupational activity (active; inactive); years of schooling (<7; 8–11; 12; +13 years); birth weight (extremely low <1,000 gr; very low 1,000–1,499 gr; low 1,500–2,499 gr, and normal >2,500 gr). Finally, gestational age was categorized as follows: extremely preterm (< 28 weeks); very preterm (28–31 weeks); moderate to late preterm (32–36 weeks) and full term (> 36 weeks). S1 Table summarizes the distribution (including missing values) of infant deaths and live births according to independent variables in periods 1990–1993 and 2014–2016.

### Risk of deaths variation

A time series analysis, using a joinpoint regression model (Joinpoint regression program, version 4.6.0.0. Statistical Research and Applications Branch, National Cancer Institute; 2018) was used for each of the U5MR components in Chile from 1990 to 2016 to describe the variation in the risk of death [18]. The response variable was the natural logarithm of each rate over time period. The selected models identify points where a statistically significant change over time in linear slope of the trend occurred. Homoscedasticity and an autocorrelation parameter were assumed. The results provide estimates of the annual percent change (APC) and its 95% Confidence Interval (95%CI).

### Cause of death analysis

The cause of death was coded using the International Classification of Diseases (ICD): ICD-9 (used from 1990–1996) and ICD-10 (from 1997 onwards), thus performing the homologation [19]. As suggested by the World Health Organization (WHO), under five deaths were divided into two age groups: Neonatal and 1–59 months. Deaths were grouped according to the respective WHO first-level categories for analysis. This distribution considers three categories: I. communicable diseases, maternal, perinatal, and nutritional conditions; II. noncommunicable diseases; and III. injuries. For this study, deaths coded as “symptoms, signs and ill-defined conditions” (780–799 in ICD-9; R00-R99 in ICD-10) were maintained as a subcategory and were not distributed proportionately to all causes, as proposed by WHO [19].

### Determinants of infant mortality

A descriptive analysis of yearly IMR according to mother’s and child factors was performed. In the case of the mother, the analysis considered age, occupational activity, marital status and schooling, as for the live birth, weight and gestational age were included. Additionally, bivariate and inequality analyses at the individual level were performed considering three-year periods, starting in 1990–1992 and ending in 2014–2016.

The bivariate analysis for infant mortality and its components were conducted using logistic regression to estimate of the probability of an infant dying, with the dependent variable being “deceased infant” (0 = No; 1 = Yes), and including as independent variables, the infant characteristics at birth and the sociodemographic conditions of the mother. For this purpose, a unique database was constructed, including all recorded live births (approximately 6.9 million) and deaths (67,681 infants) in the country for the 27-year period. The variables for the two original databases were homologated. The deceased children were included in the two databases, but as there was no unique identifier for these infants, it was not possible to recognize them. Thus, the death cases were duplicated in the new database.

Health inequality was estimated to describe the difference among socioeconomic groups of the population for infant mortality and its components. Mother´s schooling was used as the stratification variable because it was the most accurate socioeconomic variable available in the database for under one-year-old deaths. Two approaches were used for inequality analysis: simple and complex measures [20] as shown in Table 1. Statistical analyses were performed using SPSS V25 and STATA V14 with a 95% confidence interval (CI) and a p<0.05 to test the significant association.

**Table 1:** Approaches used for inequality analysis of infant mortality.

Approach	Indicator	Formula	Interpretation
Simple measures	Rate ratio of infant mortality according to mother schooling categories	It is the ratio between the child mortality in the less educated mother group against the child mortality in the most educated mother group	The value 1 means there is equality. The further the value from 1, the higher the level of inequality
		*Rate ratio = Y*_*high*_ */ Y*_*low*_	
	Risk difference of infant mortality between the extreme mother schooling categories	It is the subtraction between the incidence rate of less educated mothers and the incidence rate of more educated mothers	The risk difference takes only positive values. The larger the absolute value, the higher of level of inequality that can be attributed to mother schooling
		*Risk difference = Y*_*high*_*—Y*_*low*_	
Complex measures	Concentration curve of infant mortality according to years of mother schooling	The curve is a XY graph. In the X-axis the mothers are ranked according to their years of schooling. In the Y-axis represent the cumulative fraction of infant deaths	The 45-degree line represents the equality line. If the concentration curve lies above the equality line indicates a concentration of infant deaths among the less educated mothers. In contrast, if the curve lies over the equality line indicates a concentration of deaths among the more educated mothers
	The concentration index of infant mortality according to years of mother schooling	This index is twice the area that remains between the equality line and the concentration curve [21]	Its value ranges from -1 to 1.
			While the 0 represents equality, a negative value represent pro-poor inequality and a positive value pro-rich inequality

Source: Based on [20].

### Compliance with ethical standards

The present study follows the ethical standards of the Universidad del Desarrollo Institutional Research Committee and the 1964 Declaration of Helsinki and its later amendments. Specifically, the mortality databases used in this study do not contain any information that may identify the research subjects, and all data are publicly available from the Department of Statistics and Health Information of the Ministry of Health in Chile. Informed consent was not required, as the study involved anonymized records and datasets available in the public domain.

## Results

### Risk of death variation 1990–2016 - Under five mortality trends

Between 1990 and 2016, the U5MR in Chile underwent a 59.3% reduction, from 19.9 to 8.1 deaths per 1,000 live births, and the yearly number of under five deaths dropped from 5,813 to 1,879. Among the components of U5MR, the highest decline was in post-neonatal mortality rate (77.3% reduction), and the lowest decline was in the late neonatal rate (38.9%). The relative importance of mortality early after birth has grown; currently, 50.6% of the total under five deaths occur in the first 6 days of life (early neonatal mortality), while only 13% occur between 1–4 years old. These figures were 35.7% and 15.4%, respectively, in 1990, at the beginning of the studied period. In parallel, the birth rate decreased in Chile from 22.17 to 12.87 per 1,000 inhabitants, and the total number of live births fell from 292,145 in 1990 to 231,749 in 2016 (S2 Table).

**Fig 2:**
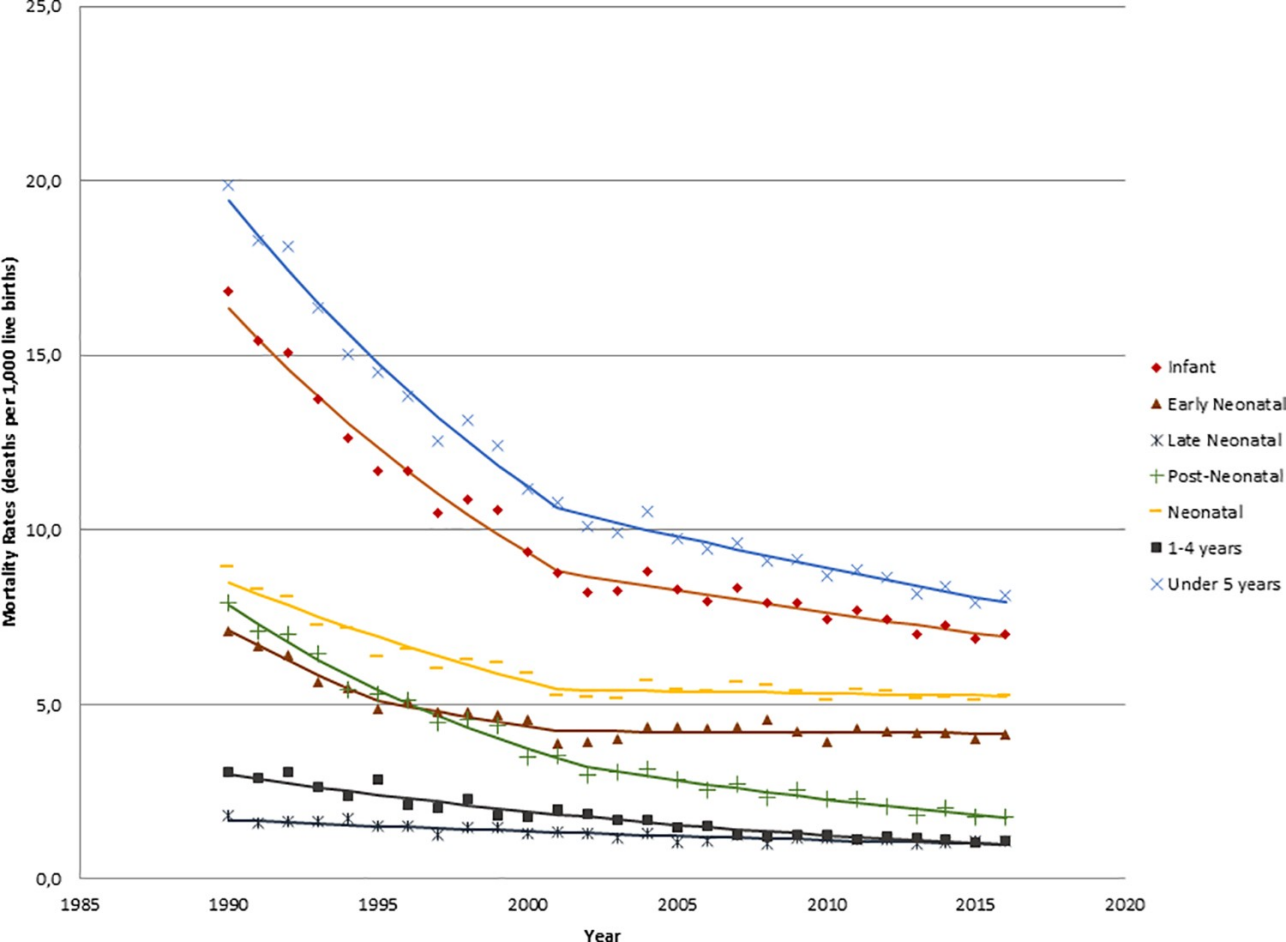
Trends of under five mortality rate and its components, Chile 1990–2016.

The trend analysis (Fig 2) produced negative slopes for all components of U5MR but with different dynamics. Table 2 illustrates the Joinpoint regression analysis for the U5MR; the far-right column shows the annual percent change (APC) of the mortality rate and the periods with significant changes in mortality rate trends.

**Table 2:** Annual percent change for under five mortality rate and its components, Chile 1990–2016.

Age group				Mortality rate per 1,000 live births		Tendency	
				1990	2016	Years	APC (95% CI)
**Under five (0–59 months)**				19.9	8.1	1990–2001	-5.3* (-5.8; -4.9)
						2001–2016	-1.9* (-2.2; -1.7)
	1–4 years (12–59 months)			3.1	1.1	1990–2016	-4.2* (-4.5; -3.9)
	Infant (0–12 months)			16.8	7.0	1990–2001	-5.4* (-6.0; -4.8)
						2001–2016	-1.6* (-2.0; -1.2)
		Neonatal (0–27 days)		8.9	5.2	1990–2001	-4.0* (-4.7; -3.3)
						2001–2016	-0.2 (-0.7; 0.2)
			Early neonatal (0–6 days)	7.1	4.1	1990–1995	-6.5* (-8.9; -4.1)
						1995–2001	-3.0* (-5.5; -0.5)
						2001–2016	-0.1 (-0.6; 0.4)
			Late neonatal (7–27 days)	1.8	1.1	1990–2016	-2.0* (-2.3; -1.7)
		Post-neonatal (1–12 months)		7.9	1.8	1990–2002	-7.1* (-7.7; -6.5)
						2002–2016	-4.2* (-4.7; -3.7)

APC, annual percent change; CI, confidence interval.

*Estimated APC is significantly different from zero at the alpha 0.05 level.

The Joinpoint model found a steady decline of the death rate in children aged 1–4 years, with an APC of -4.2. On the other hand, for IMR the model identifies two stages: a rapid decline between 1990 and 2001, with an APC of -5.4, followed by a period of slower decline (2001–2016 APC -1.6). This slowdown in the IMR decrease was related to changes in early neonatal mortality, which, after an accelerated reduction experienced a marked stagnation from 2001. The post-neonatal mortality declines also softened from 2002 but it continued dropping. All of the above led to a significant deceleration of the U5MR trend in the last 15 years, from -5.3 APC in the period 1990–2001 to -1.9 APC in the period 2001–2016.

### Cause of death analysis—neonates and children aged 1–59 months

Between 1990 and 2016, the improvement in child survival modified the distribution and the risk for specific causes of death for both groups neonates and children aged 1–59 months. In 1990 the two main causes of deaths in neonates were prematurity followed by congenital abnormalities; by 2016 the two main causes of deaths remained the same, but in inverted order. In parallel, the mortality rate fell for most of the specific causes of death in the 1990–2016 period. Injuries and acute respiratory infection experienced the highest risk reduction (98% and 95%, respectively), prematurity and sepsis also fell more than 40%. However, the group of communicable, perinatal, and nutritional causes of deaths persisted as leading causes of death among neonates, sustained by preterm birth complications and intrapartum-related events (Table 3).

**Table 3:** Neonatal causes of death, Chile 1990–2016.

Cause Category	1990			2016			Mortality rate ratio
	(292,139 live births)			(231,160 live births)			
	Number of deaths	Mortality fraction	Rate /1,000 live births	Number of deaths	Mortality fraction	Rate /1,000 live births	
							2016/1990
***All causes***	***2*,*608***	***100*.*0%***	***8*.*93***	***1*,*213***	***100*.*0%***	***5*.*25***	***0*.*59***
** I Communicable, perinatal & nutritional**	**1,751**	**67.1%**	**5.99**	**666**	**54.9%**	**2.88**	**0.48**
** **HIV/AIDS	0	-	-	0	-	-	-
** **Diarrheal diseases	2	0.1%	0.01	0	-	-	0.00
** **Pertussis	0	-	-	1	0.1%	0.004	na
** **Tetanus	0	-	-	0	-	-	-
** **Measles	0	-	-	0	-	-	-
** **Meningitis/Encephalitis	9	0.3%	0.03	3	0.2%	0.01	0.42
** **Malaria	0	-	-	0	-	-	-
** **Acute respiratory infections	146	5.6%	0.50	6	0.5%	0.03	0.05
** **Prematurity	909	34.9%	3.11	406	33.5%	1.76	0.56
** **Birth asphyxia and birth trauma	411	15.8%	1.41	87	7.2%	0.38	0.27
** **Sepsis and other RN conditions	165	6.3%	0.56	68	5.6%	0.29	0.52
** **Others group I	109	4.2%	0.37	95	7.8%	0.41	1.10
** II Noncommunicable diseases**	**716**	**27.5%**	**2.45**	**525**	**43.3%**	**2.27**	**0.93**
** **Congenital abnormalities	705	27.0%	2.41	516	42.5%	2.23	0.92
** **Others group II	11	0.4%	0.04	9	0.7%	0.04	1.03
** III Injuries**	**108**	**4.1%**	**0.37**	**2**	**0.2%**	**0.01**	**0.02**
** Symptoms, signs and ill-defined conditions**	**33**	**1.3%**	**0.11**	**20**	**1.6%**	**0.09**	**0.77**

**Fig 3:**
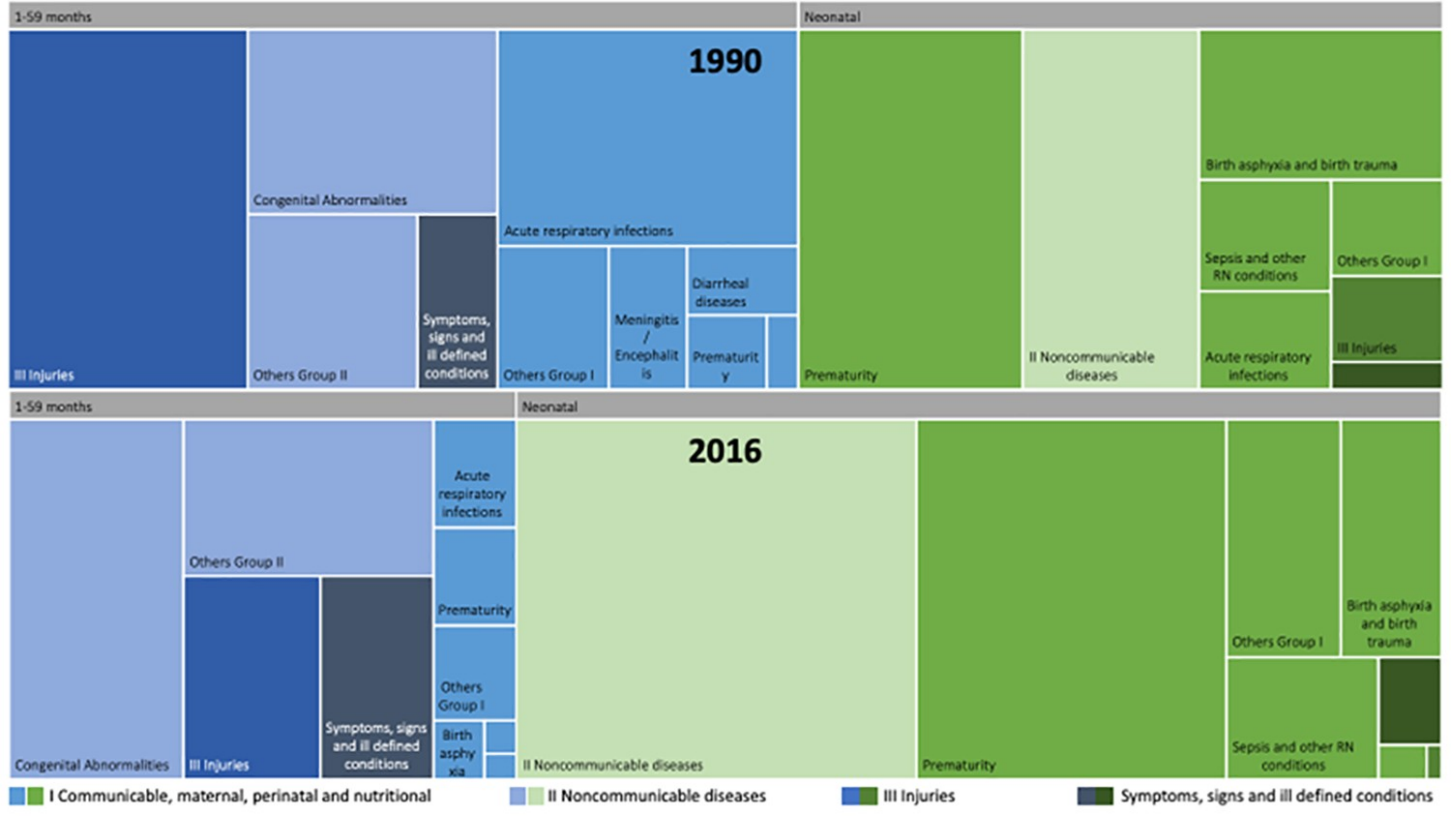
Under five causes of death distribution in Chile: Neonates vs 1–59 months. Comparison between 1990 and 2016.

In children aged 1–59 months (Table 4), congenital abnormalities persisted as the leading cause of death (34%), followed by other noncommunicable diseases (21%; mostly neoplasms, and diseases of the nervous system) and injuries (14%). Acute respiratory infection, meningitis, diarrheal diseases, injuries, and sepsis fell from 94% to 75%. Preterm birth complications and intrapartum-related events also declined by more than 40%, as did most noncommunicable diseases, including congenital abnormalities. HIV and pertussis were the only causes that increased, but they represented less than 2% of the total deaths of this group. Consequently, noncommunicable diseases (Group II) were the leading cause of death group for children aged 1–59 months in Chile during this period (Fig 3).

**Table 4:** Child aged 1–59 months causes of death, Chile 1990–2016.

Cause Category	1990			2016			Mortality rate ratio
	(292,139 live births)			(231,160 live births)			
	Number of deaths	Mortality fraction	Rate /1,000 live births	Number of deaths	Mortality fraction	Rate /1,000 live births	
							2016/1990
***All causes***	***3*,*205***	***100*.*0%***	***10*.*97***	***666***	***100*.*0%***	***2*.*88***	***0*.*26***
** I Communicable, perinatal & nutritional**	**1,220**	**38.1%**	**4.18**	**110**	**16.5%**	**0.48**	**0.11**
** **HIV/AIDS	0	-	-	1	0.2%	0.004	n/a
** **Diarrheal diseases	88	2.7%	0.30	3	0.5%	0.01	0.04
** **Pertussis	1	0.0%	0.003	5	0.8%	0.02	6.32
** **Tetanus	0	-	-	-	-	-	-
** **Measles	0	-	-	-	-	-	-
** **Meningitis/Encephalitis	126	3.9%	0.43	4	0.6%	0.02	0.04
** **Malaria	0	-	-	-	-	-	-
** **Acute respiratory infections	734	22.9%	2.51	33	5.0%	0.14	0.06
** **Prematurity	67	2.1%	0.23	30	4.5%	0.13	0.57
** **Birth asphyxia and birth trauma	26	0.8%	0.09	11	1.7%	0.05	0.53
** **Sepsis and other RN conditions	5	0.2%	0.02	1	0.2%	0.004	0.25
** **Others group I	173	5.4%	0.59	22	3.3%	0.10	0.16
** II Noncommunicable diseases**	**858**	**26.8%**	**2.94**	**372**	**55.9%**	**1.61**	**0.55**
** **Congenital abnormalities	525	16.4%	1.80	229	34.4%	0.99	0.55
** **Others group II	333	10.4%	1.14	143	21.5%	0.62	0.54
** III Injuries**	**969**	**30.2%**	**3.32**	**101**	**15.2%**	**0.44**	**0.13**
** Symptoms, signs and ill-defined conditions**	**158**	**4.9%**	**0.54**	**83**	**12.5%**	**0.36**	**0.66**

### Determinants of infant mortality

**Fig 4:**
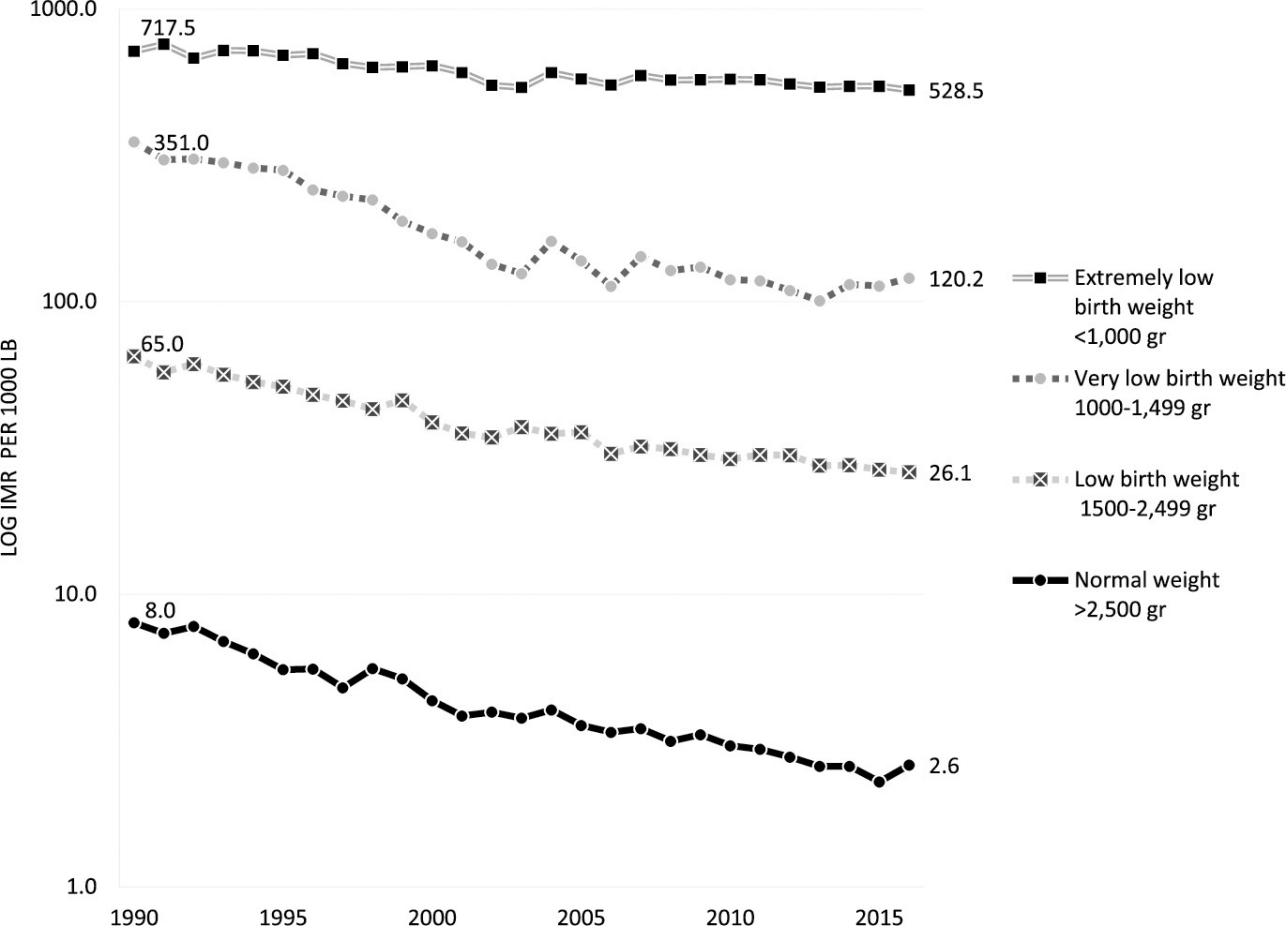
Infant mortality rate according to birth weight, Chile 1990–2016.

Live births below 2,500 gr represented 6.3% in 2016, the percentage is 10% higher compared to 1990; birth weights below 1,000 gr increased 34.8% in the same period. There was a significant variation of the IMR according to birth weight: IMR in live births over 2,500 gr was as low as 2.6/1,000 live births and was escalated ten-fold for low birth weight, 46-fold for very-low birth weight, and more than two hundred-fold for live births under 1,000 gr of birth weight. In the latter group, the IMR surpassed 500 deaths/1,000 live births (Fig 4 and S3 Table). Almost all categories of birth weight mortality risk dropped by at least half between 1990 and 2016, except for extremely low birth weight, which experienced a modest 26% reduction.

Infant mortality, according to gestational age, showed a similar dynamic. The IMR for full-term pregnancies was below 3/1,000 live births in 2016, while it rose more than 200-fold in pregnancies of fewer than 28 weeks. Almost all categories decreased their death risk by more than 60%, except for extremely preterm pregnancies, which were reduced by 19%. (S4 Table).

Concerning maternal factors, the number of mothers aged 20 or lower decreased by half between 1990 and 2016, while mothers aged 40 and older increased by 73%. In 2016, those age groups represented 11% and 4% of live births, respectively. The IMR dropped in children from all mother's age groups, showing rates of 9.4, 6.4 and 14.1 deaths/1,000 live births for mothers younger than 20 years, 20–39 years, and 40 years of age and over in 2016, respectively. Also, IMR dropped across all other sociodemographic characteristics of mothers (S5 Table).

Years of schooling, and occupational activity increased in mothers of live births during the 1990–2016 period (from 9.74 to 12.9 in years of schooling; and from 18% to 50% for occupation). The IMR dropped in all categories of the previously mentioned variables (S6 Table).

The bivariate analysis (Table 5) identified birth weight and gestational age as the two most relevant risk factors of all the analyzed infant mortality components, especially for early neonatal mortality; the more extreme the prematurity and the lower the birth weight, the higher the risk. Both risk factors increased the rate of mortality between 1990–1992 and 2014–2016.

**Table 5:** Bivariate analysis of infant mortality according to maternal and child factors, Chile 1990–2016.

		Infant mortality		Early neonatal mortality (<7 days)		Late neonatal mortality (7–27 days)		Post-neonatal mortality (28–364 days)	
Variables	Categories	1990–1992	2014–2016	1990–1992	2014–2016	1990–1992	2014–2016	1990–1992	2014–2016
		OR (95% CI)	OR (95% CI)	OR (95% CI)	OR (95% CI)	OR (95% CI)	OR (95% CI)	OR (95% CI)	OR (95% CI)
Sex (ref: female)	Male	1.20***	1.17***	1.21***	1.27***	1.16**	1.09	1.20***	1.02
		(1.16–1.24)	(1.11–1.24)	(1.14–1.27)	(1.18–1.36)	(1.04–1.28)	(0.95–1.25)	(1.14–1.26)	(0.92–1.14)
Birth weight (ref: Normal, > = 2,500)	Extremely low (< 1,000)	93.26***	216.71***	263.23***	508.12***	77.35***	199.21***	10.84***	28.19***
		(87.17–99.77)	(201.28–233.33)	(242.88–285.29)	(458.51–563.09)	(64.85–92.26)	(168.24–235.89)	(8.96–13.11)	(23.23–34.2)
	Very low (> = 1,000 gr < 1,500)	41.70***	46.5***	99.78***	89.13***	48.39***	54.98***	11.17***	15.29***
		(39.09–44.49)	(42.01–51.46)	(91.9–108.34)	(77.69–102.24)	(41.12–56.94)	(43.81–69)	(9.74–12.83)	(12.25–19.08)
	Low (> = 1,500 gr < 2,500)	7.95***	10.77***	14.2***	18.88***	7.05***	8.01***	4.98***	6.27***
		(7.59–8.34)	(9.95–11.65)	(13.23–15.25)	(16.81–21.2)	(6.11–8.13)	(6.48–9.91)	(4.64–5.36)	(5.49–7.16)
Gestational age (ref: Full term, > = 37 weeks)	Extremely preterm (<28 weeks)	103.54***	242.89***	291.92***	560.1***	62.15***	210.30***	9.98***	26.14***
		(95.86–111.83)	(224.63–262.64)	(267.26–318.86)	(505.19–620.97)	(49.76–77.62)	(175.31–252.28)	(7.85–12.69)	(20.85–32.77)
	Very preterm (28–31 weeks)	37.66***	36.65***	85.36***	65.55***	42.91***	46.73***	10.7***	12.76***
		(35.34–40.13)	(33.25–40.4)	(78.71–92.57)	(57.46–74.78)	(36.57–50.36)	(37.54–58.16)	(9.35–12.24)	(10.35–15.74)
	Moderate to late preterm (32–36 weeks)	7.64***	7.12***	13.81***	11.67***	6.73***	6.25***	4.48***	4.14***
		(7.27–8.02)	(6.58–7.71)	(12.85–14.83)	(10.4–13.09)	(5.81–7.79)	(5.07–7.71)	(4.14–4.85)	(3.61–4.75)
Mother's age (ref: 20–39 years)	< 20 years	1.51***	1.32***	1.25***	1.24***	1.61***	1.46***	1.74***	1.41***
		(1.44–1.57)	(1.22–1.43)	(1.17–1.34)	(1.11–1.39)	(1.42–1.83)	(1.2–1.79)	(1.64–1.85)	(1.21–1.65)
	40 + years	1.52***	2.07***	1.26**	2.03***	2.04***	2.21***	1.65***	2.06***
		(1.37–1.68)	(1.86–2.29)	(1.07–1.48)	(1.78–2.33)	(1.56–2.67)	(1.71–2.85)	(1.43–1.91)	(1.69–2.52)
Mother's occupational activity (ref: active)	Inactive	1.67***	1.27***	1.32***	1.12**	1.79***	1.29***	2.11***	1.66***
		(1.58–1.75)	(1.2–1.34)	(1.23–1.42)	(1.05–1.21)	(1.53–2.1)	(1.12–1.49)	(1.95–2.29)	(1.49–1.86)
Mother's marital status (ref: married)	Single	1.46***	0.52***	1.21***	0.52***	1.49***	0.50***	1.73***	0.54***
		(1.41–1.51)	(0.49–0.55)	(0.15–1.27)	(0.48–0.56)	(1.34–1.65)	(0.43–0.57)	(1.65–1.82)	(0.48–0.60)
Mother's schooling (ref: 13+ years)	1–7 years	2.76***	1.99***	1.74***	1.50***	2.67***	1.65**	4.40***	3.84***
		(2.58–2.96)	(1.74–2.28)	(1.57–1.92)	(1.24–1.81)	(2.15–3.3)	(1.15–2.38)	(3.92–4.93)	(3.06–4.82)
	8–11 years	1.92***	1.62***	1.43***	1.34***	1.93***	1.53***	2.69***	2.55***
		(1.79–2.06)	(1.5–1.74)	(1.3–1.58)	(1.22–1.48)	(1.56–2.38)	(1.26–1.86)	(2.4–3.02)	(2.2–2.96)
	12 years	1.45***	1.32***	1.34***	1.18***	1.61***	1.32***	1.58***	1.76***
		(1.35–1.56)	(1.23–1.41)	(1.21–1.48)	(1.08–1.28)	(1.29–2)	(1.12–1.56)	(1.4–1.79)	(1.53–2.02)

* p-value<0.05

**p-value<0.01

***p-value<0.001.

In the case of schooling, the steeper gradient was also clear. Higher risk was continually associated with less educated mothers and it is more relevant for post-neonatal mortality than neonatal. However, a decrease was observed across all schooling levels between 1990–1992 and 2014–2016. Other characteristics of the mother, such as inactivity (occupational activity) and age under 20 or over 40, were also positively associated with infant mortality and its components. In the case of the marital status of the mother, being single was significantly associated with a higher risk for all infant mortality components in 1990–1992, but this association was reversed by 2014–2016.

Finally, regarding the sex of the newborn, being male was significantly associated with a higher risk for all the components in the period 1990–1992 but was not significant for late neonatal mortality and post-neonatal mortality in the period 2014–2016.

### Infant mortality inequality analysis

Table 6 illustrates the IMR inequality according to the mothers’ education groups, using the measures of mortality rate ratio and risk difference. The data across education groups demonstrate a gradient pattern expressed as the rate ratio between the extreme groups. However, the IMR inequality has experienced a steady decline. The IMR decreased across all mother´s education groups between 1990–1992 and 2014–2016, and the rate ratio between child to mothers from extreme educational groups also narrowed by 27.9%, from 2.8 to 2.0. The IMR gap reduction benefited all infant death age groups: early, late neonates, and post-neonatal deaths, but with different dynamics. Early neonatal mortality shows less inequality during the observation period but has experienced modest progress (13.7% reduction). Late neonatal mortality experienced the highest gap reduction, due exclusively to the drop in the mortality of the less educated group (37.0%). Finally, post-neonatal death maintained the highest inequalities and the least progress, with only a 12.6% rate ratio reduction.

**Table 6:** Infant mortality rate and its components by years of the mothers’ schooling in Chile. Three-year period comparison (1990–1992 to 2014–2016).

Three-year period	INFANT MORTALITY				EARLY NEONATAL MORTALITY				LATE NEONATAL MORTALITY				POST-NEONATAL MORTALITY			
	Mother schooling		Rate ratio	Risk difference	Mother schooling		Rate ratio	Risk difference	Mother schooling		Rate ratio	Risk difference	Mother schooling		Rate ratio	Risk difference
	< = 7 years	> = 13 years			< = 7 years	> = 13 years			< = 7 years	> = 13 years			< = 7 years	> = 13 years		
1990–1992	22.1	8.0	**2.8**	**14.1**	7.6	4.4	**1.7**	**3.2**	2.3	0.9	**2.7**	**1.4**	12.2	2.8	**4.4**	**9.5**
1993–1995	18.3	6.8	**2.7**	**11.5**	6.0	3.5	**1.7**	**2.5**	2.3	1.0	**2.3**	**1.3**	9.9	2.3	**4.4**	**7.7**
1996–1998	15.7	6.2	**2.5**	**9.5**	5.3	3.4	**1.5**	**1.8**	2.1	0.9	**2.4**	**1.2**	8.3	1.8	**4.5**	**6.4**
1999–2001	13.5	5.7	**2.4**	**7.8**	4.9	3.3	**1.5**	**1.6**	2.0	0.9	**2.2**	**1.1**	6.7	1.5	**4.3**	**5.1**
2002–2004	12.4	5.4	**2.3**	**7.1**	5.0	3.1	**1.6**	**1.9**	1.7	0.8	**2.1**	**0.9**	5.7	1.4	**4.0**	**4.3**
2005–2007	12.0	5.5	**2.2**	**6.5**	5.2	3.4	**1.5**	**1.8**	1.6	0.8	**2.1**	**0.9**	5.2	1.4	**3.8**	**3.8**
2008–2010	11.6	5.7	**2.0**	**5.9**	4.9	3.5	**1.4**	**1.3**	1.7	0.9	**1.9**	**0.8**	5.0	1.3	**3.9**	**3.7**
2011–2013	11.1	5.7	**1.9**	**5.4**	4.9	3.7	**1.3**	**1.3**	1.6	0.8	**1.9**	**0.7**	4.6	1.2	**3.9**	**3.4**
2014–2016	11.0	5.5	**2.0**	**5.5**	5.3	3.5	**1.5**	**1.8**	1.4	0.9	**1.7**	**0.6**	4.3	1.1	**3.8**	**3.2**

The risk difference of infant mortality correlated to the mother´s education has also decreased steadily through the time series (61.1% reduction), meaning that a fraction of the risk of having an infant death due to social factors, such as years of mother’s schooling, has been slightly removed during this period, especially for post-neonatal mortality.

**Fig 5:**
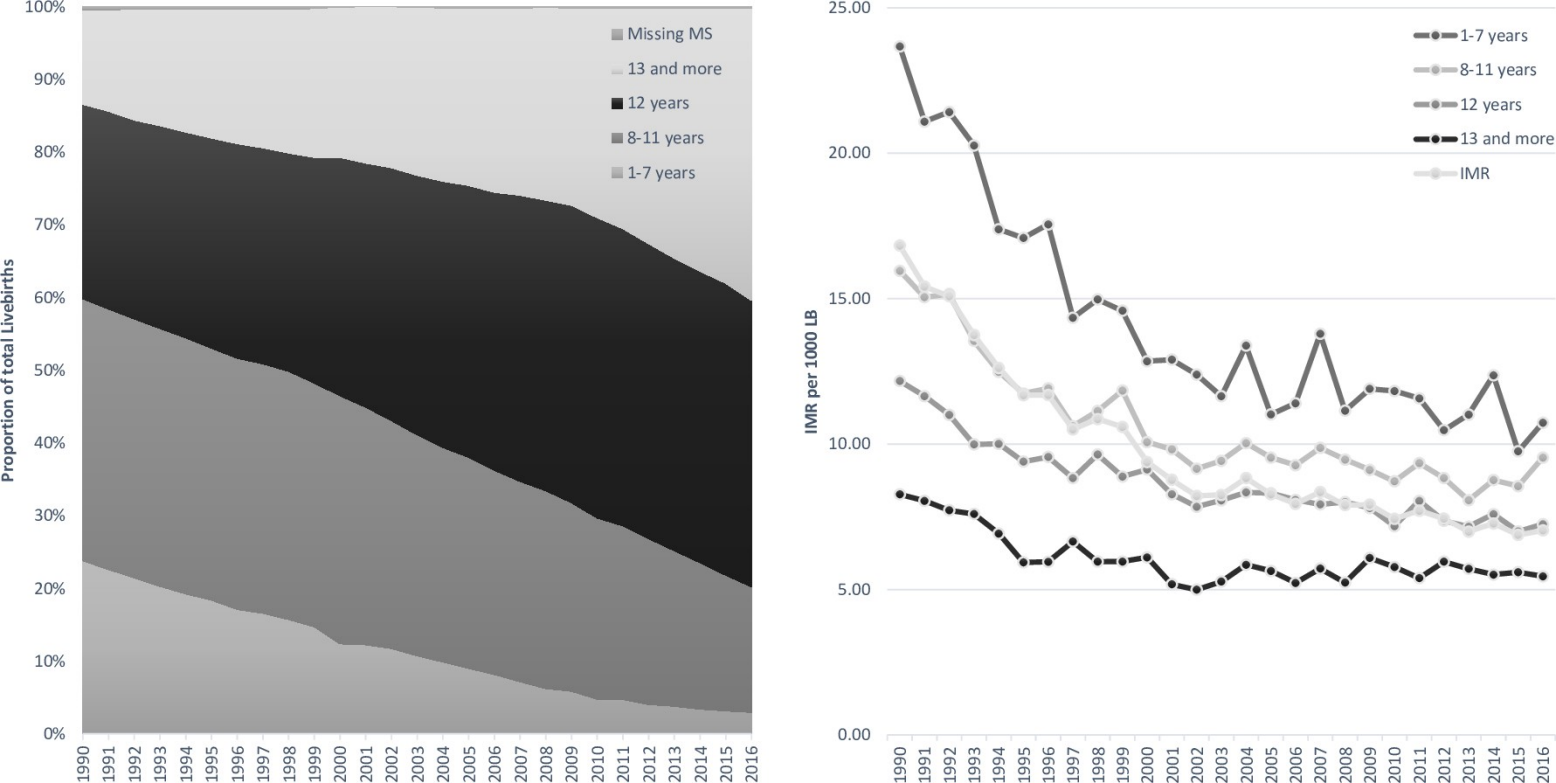
Live birth proportion and infant mortality rate according to maternal education, Chile 1990–2016.

As shown in Fig 5 the number of live births from less educated women have fallen in the period, while IMR according to mother’s schooling has also been reduced (for data details see S6 Table), which call for the need of more complex assessment measures of inequalities.

**Fig 6:**
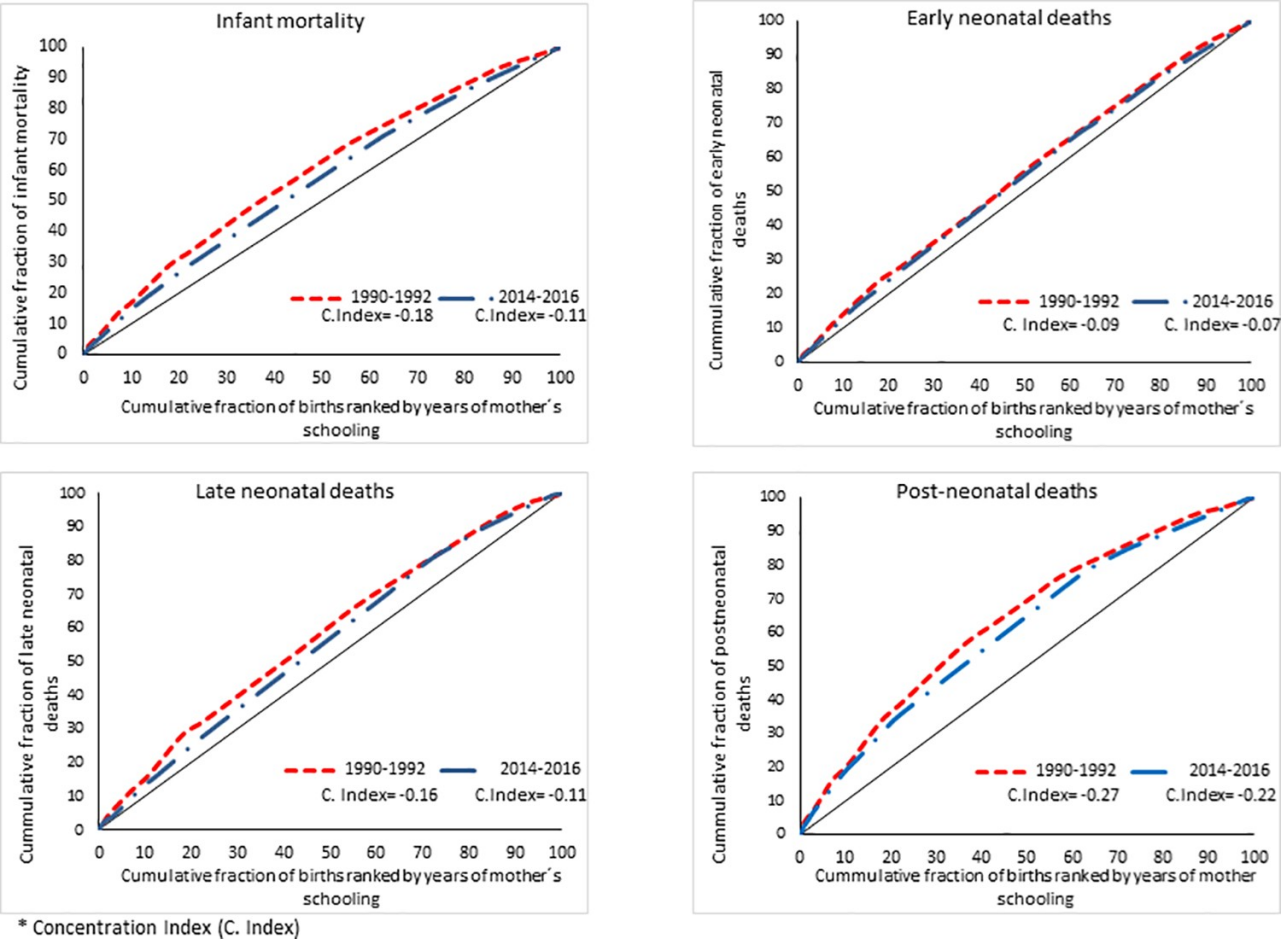
Relative inequality in the infant mortality by the mother’s schooling. Chile Triennium comparison from 1990–1992 to 2014–2016.

Fig 6 shows the concentration curve and the concentration index for the infant mortality and its components, comparing the initial 1990–1992 triennium with the last triennium (2014–2016). According to the concentration curve and index, changes over time in the relative inequality by the mother’s schooling gradient were evident in the three components of infant mortality. The relative disparity of the total infant mortality decreased 38.9% between the 1990–1992 triennium and the 2014–2016 triennium, but the inequalities remained greater in the post-neonatal deaths compared to early neonatal mortality throughout the period.

## Discussion

Child mortality has dropped significantly during the last 27 years in Chile (1990–2016). In spite of the rapid decline until 2001, there is a slower progress afterwards due to the increasing number of livebirths with extremely low birth weight. In parallel, neonatal mortality has become the main component of U5MR and congenital abnormalities the leading cause of death. The inequality of infant mortality has shown a steady decline specially in the neonatal period.

The results indicate that livebirths with extremely low birth weight is the crucial factor affecting the neonatal death dynamics in Chile. The increment in the number of very low birth weights explains the stagnation in the U5MR trend starting in 2001 in spite of the reduction in the neonatal risk of death in all the categories of birth weight. The higher frequency of low birth weight may be related to improvements of Chilean live births registries that, since 2003, include all the births regardless of weight and gestational age [22]. The more extreme the prematurity and low birth weight, the higher the risk, and both risk factors increased during the study period, or were more thoroughly reported.

The cause of death analysis showed the prominence of congenital abnormalities, surpassing prematurity, which is the first cause globally [23]. This prominence might probably change in future analysis, considering the decriminalization, since September 2017, of voluntary termination of pregnancy related to three causes, one of them being congenital abnormalities incompatible with life [22]. Acute respiratory infection was one of the few infectious causes of IMR in the 1990s, but since then it has dropped drastically. The implementation of an extended government healthy program of infectious respiratory diseases prevention through primary health care centers played an essential role in this success [6, 13]. In addition, both preterm birth complications and intrapartum-related events also declined in the context of high coverage for family planning and pregnancy care programs, especially universal access to hospital birth attendance [13].

The study provides evidence that infant mortality inequality has diminished across mother’s age, schooling, occupational activity, and marital status categories. However, the unequal distribution of infant mortality in Chile, which disproportionately affects less-educated mothers, and those who are occupationally inactive remains. Interestingly, single marital status–currently the most common condition—has no longer represent a risk factor.

The burden of education as social determinant of infant mortality is less relevant today than 27 years ago. Post-neonatal mortality dropped steadily but maintained high disparities. At the same time, early neonatal mortality has been stagnated since 2001 and appears to be highly sensitive to health interventions. The results show a cohort effect of the progress in maternal education on the IMR during the study period. The proportion of live births from less-educated mothers has fallen markedly, as has their IMR. The inequality reduction in Chile is similar to that reported by Chao and Amini Rarani [24, 25] concerning reduction in U5MR. Those researchers report absolute disparities among low- and middle-income countries and show the consequences of social policies in reducing inequalities in Iran.

The past progress in child mortality coincides with a period of unprecedented economic growth and poverty reduction that followed the recovery of democracy in Chile. Public expenditures on social programs increased significantly, including investments in the health sector that, among others, supported hospitals' infrastructure and technology, including the creation of neonatal intensive care units [6, 26]. Improvements in social determinants and expanded access to care were related to the accelerated progress observed during the 1990s and the slower but steady improvement in this century [1, 6, 26]. The 2005 health reform introduced a regimen of explicit health guarantees, known as AUGE, embracing, among other priorities, the leading causes of child mortality. All children affected by perinatal diseases, congenital cardiac anomalies, and acute respiratory infections, as priority diseases, have legally binding guarantees of access, opportunity, quality, and financial protection for their treatments [11–13]. Concerning intersectoral action, the government created in 2005 the program *“Chile Crece Contigo”*, based in the Ministry of Social Development, which consists of a comprehensive universal child protection system, that covers children from pregnancy to 9 years of age [14, 15].

The most relevant limitation of this study is the unavailability of social variables for children aged 12-59-month deaths that restricts the analysis of determinants and inequality. Considering the association between the higher age and higher inequality, it is possible to hypothesize wider inequality in the unstudied age groups. Another limitation is the lack of other social variables usually available in survey-based studies—such as poverty, migrant conditions, and ethnic background—that humpers alternative analysis. However, the infant mortality gradient by education of the mother is considered a good indicator to evaluate the impact of social inequalities in health [20]. Updating the official death certificate homologating requirements for under five deaths and completing social variables will contribute to better analyses. Additionally, it is relevant to reduce the proportion of ill-defined causes of deaths among children aged 1–59 months, which reached 12.5% in 2016.

The past evolution indicates that child health inequality can be reduced, even in an unequal country. Despite the significant progress, Chile, a high-income country since 2013, maintains a rate of U5MR that is higher than the average of the high-income group of nations, thus demanding improvements [27].

Public policies implications include targeting early mortality by improving the quality of antenatal care and access neonatal units. Congenital abnormalities require technology for early detection (advanced diagnostic imaging) and complex treatments that are currently either not offered or not covered by health insurance (public and private) in Chile. Regarding inequality, emphasizing universal access and intersectoral interventions are needed to continue addressing the social determinants affecting the more vulnerable groups.

In conclusion, the Chilean experience illustrates excellent child health achievements but remaining serious challenges in a country that has transitioned from middle- to high-income in recent decades.

## Supporting information

S1 Table Distribution of infant deaths and live births according to independent variables, periods 1990–1993 and 2014–2016. (XLSX)

S2 Table Under five mortality rate and its components, Chile 1990–2016. (XLSX)

S3 Table Infant mortality rate trend according to birth weight, Chile 1990–2016. (XLSX)

S4 Table Infant mortality rate according to gestational age, Chile 1990–2016. (XLSX)

S5 Table Infant mortality trend according to maternal factors: Age, occupational activity and marital status. (XLSX)

S6 Table Infant mortality according to mother's schooling, Chile 1990–2016. (XLSX)
